# Grasping at digitalisation: turning imagination into fact in the sugarcane farming community

**DOI:** 10.1007/s11625-020-00885-9

**Published:** 2021-01-02

**Authors:** Simon J. Fielke, Bruce M. Taylor, Emma Jakku, Martijn Mooij, Cara Stitzlein, Aysha Fleming, Peter J. Thorburn, Anthony J. Webster, Aaron Davis, Maria P. Vilas

**Affiliations:** 1 CSIRO Land and Water, Dutton Park, Australia; 2 CSIRO Data 61, Fortitude Valley, Australia; 3 CSIRO Data 61, Sandy Bay, Australia; 4 CSIRO Land and Water, Hobart, Australia; 5 CSIRO Agriculture and Food, Saint Lucia, Australia; 6 CSIRO Agriculture and Food, Cairns, Australia; 7 James Cook University, Townsville, Australia; 8 DNRME, Queensland Government, Dutton Park, Australia

**Keywords:** Digital technology, Social science, User experience, Responsible innovation, Agriculture

## Abstract

Nutrient runoff from catchments that drain into the Great Barrier Reef (GBR) is a significant source of stress for this World Heritage Area. An alliance of collaborative on-ground water quality monitoring (Project 25) and technologically driven digital application development (Digiscape GBR) projects were formulated to provide data that highlighted the contribution of a network of Australian sugar cane farmers, amongst other sources, to nutrient runoff. This environmental data and subsequent information were extended to the farming community through scientist-led feedback sessions and the development of specialised digital technology (1622™WQ) that help build an understanding of the nutrient movements, in this case nitrogen, such that farmers might think about and eventually act to alter their fertilizer application practices. This paper reflects on a socio-environmental sustainability challenge that emerged during this case study, by utilising the nascent concept of digi-grasping. We highlight the importance of the entire agricultural knowledge and advice network being part of an innovation journey to increase the utility of digital agricultural technologies developed to increase overall sustainability. We develop the digi-MAST analytical framework, which explores modes of being and doing in the digital world, ranging from ‘the everyday mystery of the digital world (M)’, through digital ‘awareness (A)’, digitally ‘sparked’ being/s (S), and finally the ability of individuals and/or groups to ‘transform (T)’ utilising digital technologies and human imaginations. Our digi-MAST framework allows us to compare agricultural actors, in this case, to understand present modes of digi-grasping to help determine the resources and actions likely to be required to achieve impact from the development of various forms of digital technological research outputs.

## Introduction

It has been argued that agriculture is undergoing a technology revolution (Rose and Chilvers 2018). This revolution has been labelled ‘agriculture 4.0’ after the shift from hunting and gathering to settlement, industrialisation and mechanisation, and the green revolution before it (Rose and Chilvers 2018; Rotz et al. 2019). While the technological change driven by the digitalisation of everything is said to be a catalyst for widespread environmental and economic benefits—examination of the human competencies and social learning required to plan, operate and flourish in such a future have been neglected (Eastwood et al. 2019b; Helbing 2019). Similarly, notions of responsibility permeate the development of these socio-technological futures (Gremmen et al. 2019). This paper builds on the groundwork of innovation diffusion literature (Eastwood et al. 2017; Hekkert et al. 2007; Rogers 2010) recognising that such a social process is always dynamic and multifaceted (Glover et al. 2019; Pannell and Claassen 2020). To date, academic examination in the digital technology space has been largely conceptual and lacked grounding in the real-world practice of individual technology development processes with human developers and users (Dufva and Dufva 2019; Glover et al. 2019; Higgins and Bryant 2020; Klerkx and Rose 2020). We build on work imagining the diversity of human and digital interactions to develop the digi-MAST framework to address this gap when considering future digital agricultural technological developments and the human outcomes being sought by proponents.

**Fig. 1:**
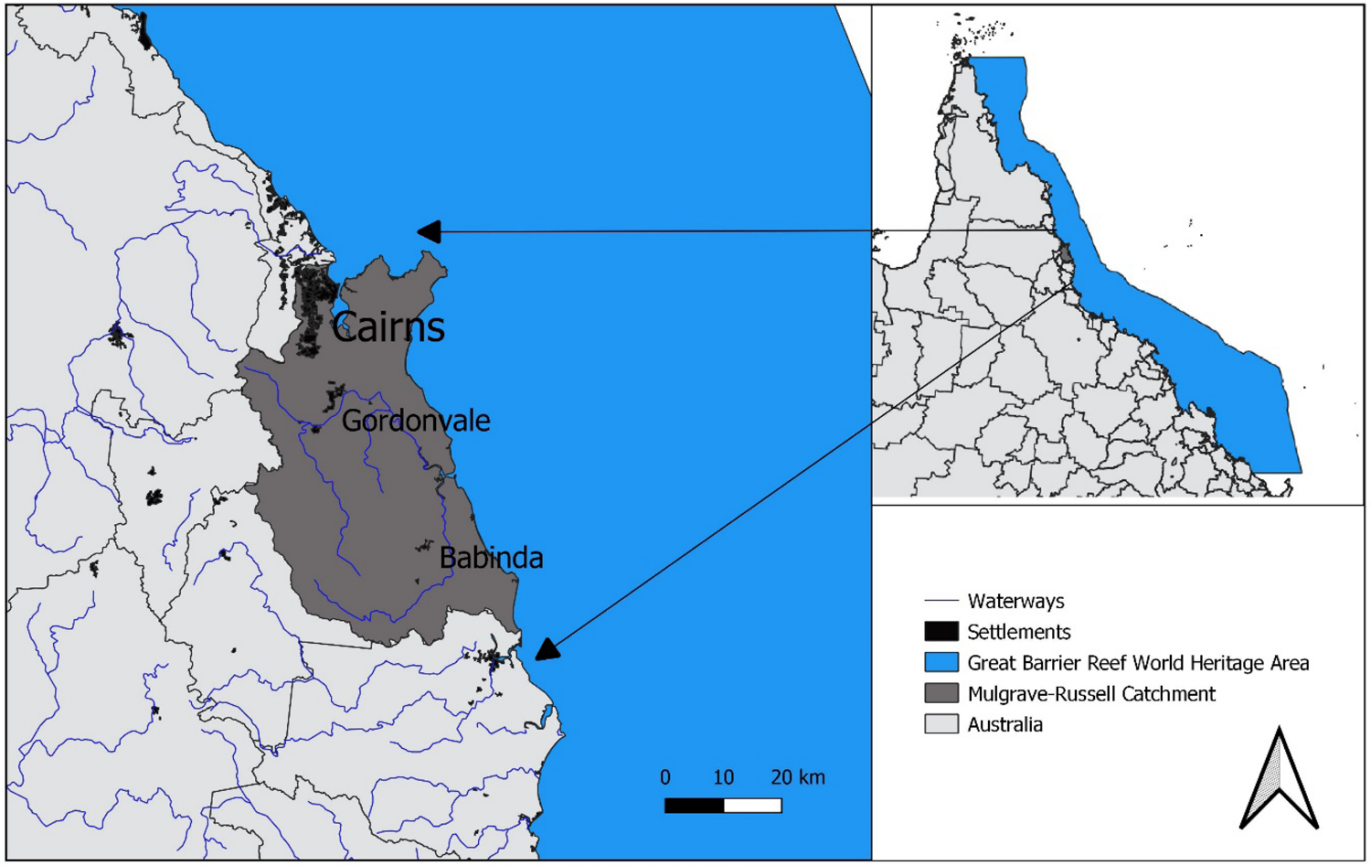
Map of the Mulgrave-Russell catchment

There is increased focus in developing digital technologies that allow farmers to maximise productivity while minimising the environmental impacts of agricultural production indicating the potential for improved sustainability outcomes (Kernecker et al. 2021; Klerkx and Rose 2020; Rose and Chilvers 2018). Nitrogen losses at the farm level are the main contributor to global nitrogen pollution (Kanter et al. 2020) and are threatening the health of aquatic ecosystems by causing degradation through eutrophication (Fowler et al. 2013). In Australia, nitrogen pollution is threatening the health of a world-heritage listed ecosystem, the Great Barrier Reef (GBR). To protect the GBR, the Government of Queensland has established the Water Quality Improvement Plan focused on optimising nutrient management by encouraging the adoption of Best Management Practices (BMPs) (Queensland Government 2018). Unfortunately, progress towards land management targets is relatively low (Taylor and Eberhard 2020). Due to the importance of sugarcane land management practice on reaching targets for dissolved inorganic nitrogen we focus on the change in thinking required to alter nitrogen fertilizer use of human stakeholders in the Australian sugarcane agricultural knowledge and advice network situated within the Mulgrave-Russell catchment in Queensland, Australia (Fig. 1). We focused on this catchment because it has been defined as a high management priority for water quality improvement (Queensland Government 2018).

Digi-grasping was recently conceptualised to denote the journey that is required to understand (grasp) the potential of digitalisation to empower people to take part in a digital society (Dufva and Dufva 2019). The modes of digi-grasping present within agricultural systems will directly influence the likelihood of successful digital technology adoption, ongoing use and iteration. In this study we applied the digi-grasping conceptualisation to develop a novel framework, which we refer to as digi-MAST, to support recent work that recognises that simple conceptualisations of technology adoption processes are problematic (Glover et al. 2019). This is in response to the need articulated by Pink et al. (2018) to deal with ‘data anxieties’ created by the ‘digital mess’. As such, we explore the following research question: how is the digi-MAST framework a valuable tool for considering the changes required to grasp new digital technologies in the Australian sugarcane farming industry? We develop the digi-MAST framework to inductively apply the concept of digi-grasping (Dufva and Dufva 2019) to the real-world perceptions of individuals and groups of human actors engaging with (or not) a specific digital technology—1622™WQ (CSIRO 2019; Vilas et al. 2020). The four modes of digi-MAST allow exploration of the status of agricultural knowledge and advice network actor perceptions in relation to specific digital technologies. The Digi-MAST framework refers to the embodied experience of the digital world by adapting four modes: the ‘everyday mystery of the digital world’ (M); ‘awareness of the digital world’ (A); ‘empowered being/s’ with interest sparked (S); and ‘transformation and aesthetics’ (T) (building on Dufva and Dufva (2019) and their digi-grasping modes). We depart from the digi-grasping conceptualisation to introduce the notion of sparked (S) rather than ‘empowerment’ as it better represented the descriptive word that farmers, advisors and researchers felt or were observed exuding. Being sparked is to go beyond the passivity of awareness and engage emotively (either willingly or sometimes unwillingly) with the technology.

The need for such a framework in the context of the Australian sugarcane farming industry helps explain why previous attempts to digitalise the industry may not have achieved their aims. For example, decision-making tools such as ‘SafeGuard’ for pesticides did not seem to gather the traction required to be adopted by the farming community (Queensland Government 2020). Combined with an assortment of emerging digital agricultural applications it is timely to reflect on the challenges and opportunities to engage agricultural knowledge and advice network actors with digital technologies in the pursuit of responsible innovation and the implications of these applications moving forward (Fielke et al. 2019). The digi-MAST framework provides an opportunity to explore these socio-digital processes with greater nuance and recognition of actor agency than the adoption curve heuristic suggests (Douthwaite et al. 2001; Rogers 2010).

## Methods

The case study explored here utilises social science conducted as part of a collaborative water quality monitoring initiative in the Mulgrave-Russell catchment of North Queensland (Fig. 1). Project 25 (P25) is a farmer-led water quality monitoring project to improve the sustainability of sugarcane farming (NESP TWQ 2016). P25 involves state-of-the-art sensing technologies and techniques to monitor water quality in this catchment and includes a process of the lead researcher communicating results back to farmers at various local engagements in an ongoing capacity approximately every few months. Simultaneously, the Digiscape Future Science Platform (FSP), a large programme of research in Australia tasked with harnessing the digital revolution for landholders was funded. One of the Digiscape FSP projects involved a use case situated within the GBR and the project team formed a collaborative alliance with P25 farmers, researchers and their institutions, to develop 1622^TM^WQ, an application for farmers to access and visualise water quality from these P25 (and other) sensors on their digital devices (CSIRO 2019). 1622™WQ provides near real-time water quality information at multiple sites within this case study catchment allowing farmers to see differences in water quality in sites located upstream or downstream from their (and others) farms [for a thorough review of this technology development process see Vilas et al. (2020)]. The hypothesis of the P25 and Digiscape GBR (DGBR) research alliance was that providing near real-time information via a digital application (1622™WQ) would help support change in thinking about nutrient movement through the catchment and perhaps then alter farmer behaviour such that they adjust nitrogen fertilizer management decisions. By providing them with information about water quality the impact of this project would work to reduce one stressor on the valuable natural wonder of the GBR, nutrient run off. Implied in this case study was an assumption that farmers might be willing to alter their behaviour because of information they could obtain through digital technology. However, it was also recognised that to explore nutrient movement through the catchment farmers would have to interact with scientists and that digital technologies could help in supporting this exploration by providing a boundary object and data display interface for farmer-scientist/advisor discussions (Jakku and Thorburn 2010).

Recognition that the implications of research projects being analysed in this case report, and others like it, rely on stakeholder engagement and change within both digital and physical worlds mean that while the digitalisation of agricultural systems holds great promise, networks of human actors will largely determine the success or otherwise of such endeavours. As such, embedded social science methodologies are required to delve into the ‘socio’ component of developments in these socio-technical systems so interviews were undertaken with actors (*n* = 20) within in the sugarcane industry knowledge and advice network (Fielke et al. 2018; King et al. 2019). This process began with interviews conducted from late 2018 following an exploratory case study methodology that has been used previously (Fielke and Srinivasan 2018; Yin 2014). These interviews were conducted either face-to-face (preferred) or via telephone (when necessary) and ranged between 40 and 90 min.^1^ The following were key overarching open-ended lines of questioning involving numerous sub-questions, with the relevance of the questions highlighted in brackets and the **most critical in relation to developing the digi-MAST framework in bold**:

- Where do you get information about farm management—Who are your key sources of information and where do you go if you have questions? (To understand context of case study agricultural knowledge and advice network)
- What is the contribution of local networks, relationships, or events in terms of your farm management decision-making? (To understand how respondents and their peers make decisions)
- Do you think anything could be done to improve water quality monitoring or its applicability to land management decisions, in your region? (To understand how the monitoring technologies were (or were not) beginning to influence respondents)
- **Are there any parts of your business that involve digital technology—Are there any parts which you see could involve digital technology in the future? (To understand the specific digital tools used and/or level of eagerness to learn about/use those tools)**
- **Have you heard of/used the 1622™WQ technology? If yes, how did you feel when interacting with it? (To understand the direct relevance of perceptions of the technology in question)**
- **Do you see changes in technology as being generally positive or generally negative in the future? (To understand how technologies influence respondent perceptions about what is (un)desirable change)**

The interviews were recorded and professionally transcribed. The interviewees were water quality, agronomic, and human-centred design researchers, farmer-based extension advisors and farmers themselves (Table 1). Most of these interviewees were involved with the development and deployment of 1622™WQ application digital technology that is attempting to monitor the quality of water flowing through various points of the Mulgrave-Russell catchment, to improve environmental outcomes for the GBR (see http://www.1622.farm). We also interviewed farmers that were not explicitly involved with either P25 or DGBR (1622™WQ) to further understand the agricultural system the project teams and technologies were deploying into. In terms of the farmers (both involved and not involved groups) interviews were conducted to the point that recurring themes were prominent (sometimes very frequently) around causes of water quality issues, use of technologies, and advice network change. Given this saturation of topics, further interviewers were not pursued.

**Table 1:** Case study interviewee codes, role and additional information/relevance to water quality (WQ) project

Interviewee code	Role	Additional information/relevance
A1	Advisor	Local industry body representative
A2	Advisor	Local extension officer
A3	Advisor	Local extension officer
R1	Researcher	Design
R2	Researcher	Water quality
R3	Researcher	Agronomy
R4	Researcher	Industry research organisation
R5	Researcher	State government
IF1	Farmer	Involved in WQ project
IF2	Farmer	Involved in WQ project
IF3	Farmer	Involved in WQ project
IF4	Farmer	Involved in WQ project
IF5	Farmer	Involved in WQ project
IF6	Farmer	Involved in WQ project
NF1	Farmer	Not involved
NF2	Farmer	Not involved
NF3	Farmer	Not involved
NF4	Farmer	Not involved
NF5	Farmer	Not involved
NF6	Farmer	Not involved

The interviews were conducted and then reported to the project team developing 1622™WQ (the specific digital technology) with a focus on the case study region and industry. The social scientists embedded in the project team then used the concept of digi-grasping to build the digi-MAST framework, thematically analyse the interview data, and then report back to the project team to explain why this was not a simple technology adoption problem considering the current agricultural system context. This was the inductive process followed to help bring social concerns raised by the farming community to the technology development team and provide a tool to understand how and why some people would not engage with their product. The results section is structured with an overview of data collected from one of these workshopping sessions (see “Workshopping qualitative findings with the digital application development team”) which then led to the inductive re-analysis of interview data to develop the digi-MAST framework (see “The everyday mystery of the digital world (M)”, “Awareness of the digital world (A)”, “A spark empowers human beings in the digital world (S)”,  “Transformation and intentional creation in the digital world (T)”).

We followed principles of responsible research ethics in the big data era by valuing diversity in various forms for example: including different interviewee perceptions and levels of project involvement (Sorrell 2018); not shying away from a contentious research space; providing nuance to the regional context; and reaching out to multiple sectoral stakeholders (Hesse et al. 2019). This project included interviews with ‘practitioner’ stakeholders at different points of the knowledge and advice network value chain (researchers, advisors and farmers that were both involved and not involved in the specific research) (Brandt et al. 2013). The use of nitrogen fertiliser and its relationship with water quality is widely and fiercely contested with regard to who is (and how much they are) to blame for negative impacts on the GBR (van Grieken et al. 2019). We provide nuance to the regional context by (where possible) embedding ongoing research interactions (for example through multiple rounds of prototyping, designing and user testing as well as formal social research methodology) within the Russell-Mulgrave region (Vilas et al. 2020).^2^ Finally, as mentioned previously and seen in Table 1 we attempted to gather perceptions through various sectoral stakeholders, although we had to make trade-offs between the number and type while achieving an effective snapshot of the agricultural system case study. We recognise that the subsequent development of the digi-MAST heuristic framework may only be applicable to this socio-technical case study. It is important, however, to start a process of qualitatively monitoring the effectiveness and value of agricultural practices that include the use of digital technologies so that justifications and reasoning for non, partial or temporary adoption can be strengthened when valid (Pannell and Claassen 2020).

## Analytical framework: Digi-MAST as a human-centred design heuristic to grasp the digital

To jump the chasm of disillusionment and to begin to deliver on the hype of a digitally enabled agricultural innovation system, various individual human actors will need to grasp digital futures (Dufva and Dufva 2019; Fielke et al. 2019). To that end, we used user experience and social science methodologies embedded in technology design cycles as part of the DGBR project meetings to develop the digi-MAST framework, whereby modes were labelled mystery (M), aware (A), spark (S), and transform (T) (for more information on this process see Sect. 4.3 and Fig. 6 in Vilas et al. (2020)). These modes are worked through in an iterative process as they relate to specific digital technologies—through individual and collective learning in the agricultural innovation system, agricultural knowledge and advice networks, and within agricultural advisory service typologies (Table 2) (Fielke et al. 2020). Importantly, while the digi-MAST framework suggests different levels of individual and collective understanding of the digital world, it is possible to operate in various, fluctuating, modes of digi-grasping when different digital technology platforms are engaged with at different times. For example, utilising a smartphone application to interact with and understand nutrient runoff measures might initially be extremely frustrating (suggesting operation in mode M). Although engaging a localised weather smartphone application may now be a common occurrence for the same stakeholder, resulting in transformation (T) whereby the nightly news or daily newspaper weather bulletins becomes far less relevant in determining farm management decision-making. As such, this paper aims to contribute to the nascent scholarship on digital technologies as an opportunity to achieve sustainability outcomes in the agricultural sector (Anastasiadis et al. 2018; Klerkx and Begemann 2020).

**Table 2:** Defining digi-grasping modes through the digi-MAST framework

	Mode	Example indicators	Description [adapted from Dufva and Dufva (2019)]
M	Mystery	I have no idea, I don’t care, I don’t see X as important, I don’t have the resources to consider X	Being and doing in the digital world is taken for granted, not acknowledged: utilisation of devices and software are done without an awareness of the influence of digital technologies. Use can be fluent and effortless or annoying and forced… but use happens without a grasp of the digital infrastructure, systems or influence on individual behaviours
A	Aware	I have heard of X, tell me more, help me understand the value of X so that I can consider it further	Becoming aware of the surrounding digitality: being conscious of digital presence in our everyday lives and interrelation with the digital world… it could be difficult to articulate the feeling of being or doing in the interface between the digital and physical, but this articulation is not necessary to grasp how digital technologies and digitalisation affect everyday life and being
S	Spark	I want to play with X! This is fun/a hobby, I’ll have a go, I’m understanding how X can fit in with my values and physical world	Going beyond awareness and shifting the focus to how things could be: The ability to grasp digitality enables one to question the relationship with the digital world… What is the interface between physical and digital worlds? Why? Can it be different? Enables the questioning of moral issues as well as a feeling of responsibility for the consequences of digitalisation
T	Transform	I am going to invest in X because it could change how I do things, I am gaining confidence, I am able to share with others	Reclaiming agency in the interface between digital and physical worlds to shape the direction of future developments: Intentional creation add aesthetic qualities, beyond moral and political questions… the digital world allows for a more intuitive and sensory experience. Aligning with what Helbing (2019) refers to as ‘digital enlightenment’

**Fig. 2:**
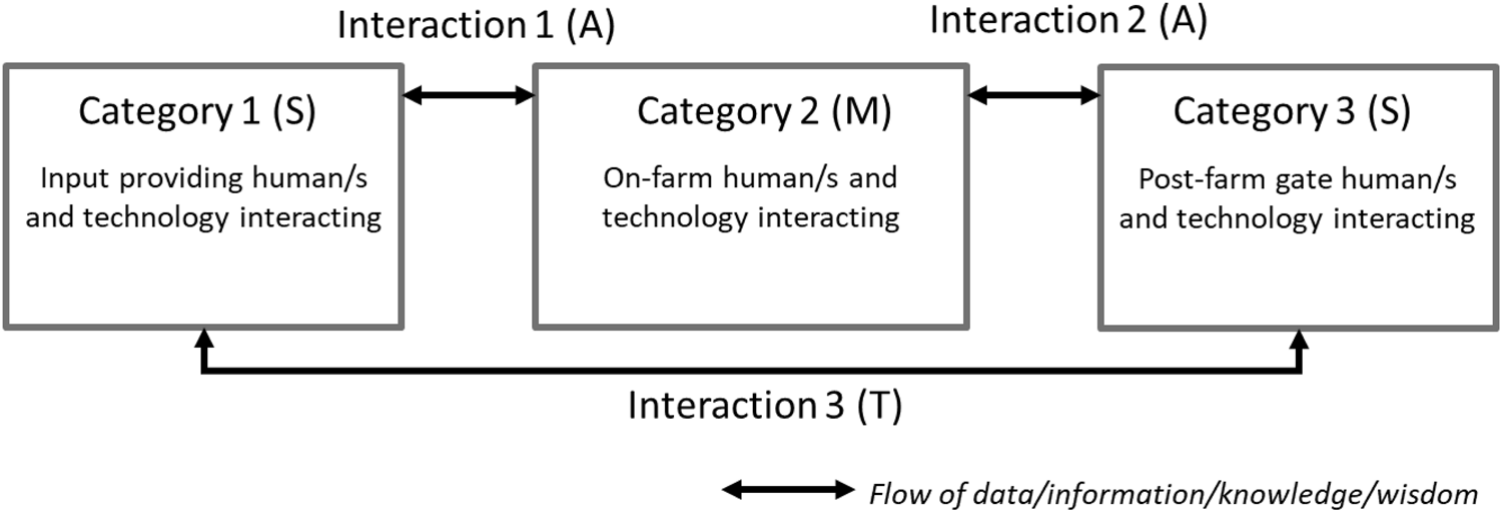
Interactions within and between components of the sugarcane agricultural knowledge and advice network (with current 1622™WQ digi-MAST mode generalisation in brackets) [Adapted from: Fielke et al. (2020)]

Figure 2 represents the inter-connection of agricultural advisory services, input and post-farmgate human and technological actors through interactions with on-farm humans and technologies (Eastwood et al. 2019a; Knierim et al. 2017). These interactions can each be represented by a spectrum of digi-MAST modes, whereby different human and technological opportunities can be realised across these modes. This framework was built considering existing data, information, knowledge, and wisdom (DIKW) theoretical flows and in the context of continual learning and development (Janssen et al. 2017). Each category and interaction in Fig. 2 represent the involvement of specific digital technologies that can be grasped by humans at one of the digi-MAST modes. The modes indicated in brackets are project team assessments in relation to the case study agricultural knowledge and advice network, P25 and Digiscape GBR human actors, and the digital technology application, 1622™WQ (Thorburn et al. 2019).

This digi-MAST framework overlays a constructivist lens, that different actors have different views of ‘reality’, to the realist digital technological development process whereby 0 s and 1 s must be coded to formulate the ‘objective’ digital worlds we create (Scholz and Steiner 2015). The digi-MAST framework recognises the importance of building individual and collective absorptive capacity—the capacity to transform modes of thinking and doing through iterative social learning (Turner et al. 2017)—through digi-grasping (Dufva and Dufva 2019). To test the utility of this framework we need to explore individual human actor relationships with specific technologies through our qualitative data.

## Results and analysis

It is important to recognise that human perceptions of technology costs and benefits change based on their engagements within and outside of their networks, and the specific the technology or suite of technologies in question (i.e. technology readiness or assessment of digital tool value) (Ayre et al. 2019; Parasuraman 2000).

### Workshopping qualitative findings with the digital application development team

**Fig. 3:**
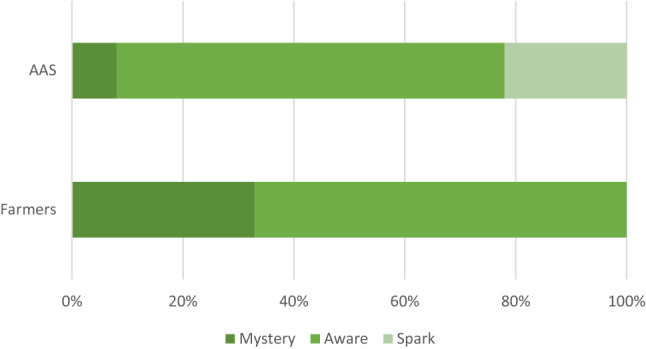
Project team perceptions of the digi-MAST mode of farmers and agricultural advisory service (AAS) stakeholders in relation to the 1622™WQ application. Note: AAS category was aggregated up from three AAS categories (public authority AAS, public research and education AAS, and private AAS—see Knierim et al. (2017) for more information)

The DGBR project team gathered for a workshop to further develop the suite of technologies under development in late 2018. At this workshop, the digi-MAST framework was introduced to better understand where the current project stakeholders were situated in relation to modes of digi-grasping and the 1622™WQ application. Figure 3 shows the results of a workshop exercise where the project team placed farmer and agricultural advisory service (AAS) stakeholders into a mode of digi-MAST based on their interactions and perceptions to date (which subsequently was found to align with the interview findings—see Fig. 2). A project team member recalled aspects of their work that aligned with a learning transition from living with the mystery of the digital world and overcoming multiple frustrations to spark their interest and learning relating to a deploying infrastructure that feeds information into the 1622™WQ application (see Box 1).

The use of the digi-MAST framework by the project team allowed for tools being developed alongside 1622™WQ to be considered against the current digi-grasp of stakeholders who would need to be interested and buy into the application to achieve envisaged impacts. More importantly, the digi-MAST framework allowed the team to look beyond the immediate project to imagine what changes might be required, and who would need to have their interest sparked for wide-spread use, for example considering the implications of increased nutrient management regulation, or economic incentives for behaviour change.

We summarise our analysis of the digi-MAST mode of the farmer and agricultural advisory service interviewee comments in Table 3. The various interactions taking place in this case study of an agricultural knowledge and advice network were also hypothesised to align with modes generalised in Fig. 2. As such the framework captures the complexity of the different actors in the networks and their interactions with digital technologies. In the remainder of the results, we utilise the digi-MAST framework to explain different human actor responses and affordances regarding technological interaction between nodes of the regionally-based case study agricultural knowledge and advice network (Berthet et al. 2018).

**Table 3:** Mode of interviewee comments in the digi-MAST framework

Digi-MAST mode	Farmers	Agricultural advisory services
Mystery	6	1 (advisors referring to farmers)
Aware	3	0
Spark	2	2
Transform	1	2

Box 1: the example of a researcher progressing through digi-MAST modes: from mystery to spark in the context of technology deployment that feeds data into 1622™WQ A DGBR team member described that through the process of ‘making’ and experimentation of setting up a system of rainfall gauges to feed data into the application (1622™WQ), they were able to explore how the digital technology turned rainfall readings into either a 1 or a 0 (Dufva and Dufva 2019). The discussion began with the team member explaining that they were implementing a process to ‘build, measure, and learn’—comprising validated learning experiments that involved an automated rainfall gauge network linked together by ‘Internet of Things’ technology. Initially, the idea was to provide real-time, localised rainfall information to farmers by uploading the data to the 1622™WQ application to increase understanding regarding the link between rainfall and nitrogen runoff. The team member found, however, that it ‘sounds easy on paper, until you go out to do it’. Their story involved many trips to both local hardware stores and online electronics resale outlets to obtain materials to test, situate and learn about how the technologies worked. The frustration of it not being ‘easy’ and an ongoing process was referred to by the project leader as the team member’s ‘hobby’, with the team member referring to it being a ‘bit of a pain but challenging and fun in reality’. The individual’s interest drove the initiative in terms of embracing challenges in both the digital and physical worlds to come up with a sensor network that is now in place for farmers to receive real-time and location specific rainfall information through 1622™WQ. Subsequently, this information is being utilised to validate another spin-off research endeavour to further increase interest in the 1622™ suite of tool features beyond water quality monitoring readings. These efforts on behalf of the individual project team member saw the original ‘mystery of the digital world’ turn into an awareness of the technological potential and ‘spark’ of the individual’s interest to get the rainfall sensor network to feed into the 1622™ set of applications.

### The everyday mystery of the digital world (M)

While we acknowledge reported heterogeneity of sugarcane farmers (van Grieken et al. 2019), we categorised those interviewed for this work according to whether or not they were involved with the P25-Digiscape collaborative water quality monitoring alliance. Interestingly, farmers from both categories (involved and not involved) indicated that they were confused by the ‘everyday mystery of the digital world’. For example, when asked how useful the development of the 1622™WQ application might be for them, responses from farmers not involved in the project included:

Well it would be of assistance if I knew how to drive a computer [indicating mystification], but I am computer illiterate [laughs]. I get it all done [by a staff member] (NF2).

The only thing for that is sometimes you might have to explain to me how it’s functioning [indicating mystification], so I understand it (NF6).

It was evident that engaging with something new and attitudes of ‘having’ to learn as opposed to ‘wanting’ to learn can be a barrier to technology experimentation. Indicating their need for upskilling and increased technological awareness before the technology would be useful to them, this line of questioning provoked the following narrative from a farmer who was involved in the project—more for water quality monitoring reasons that in terms of technological development:

They started talking about putting things on your farm and you can do this and do that. I’m lost, mate [indicating mystification]. I was beyond my depth. I’m way beyond my depth. This is my phone (shows interviewer flip phone). I’ve got three messages and one missed call. That’s all I need to know… I still can’t get my head around why everybody puts everything on [the internet] (IF4).

This mindset was supported by advisor interviews who recognised the challenge in convincing certain farmers that there might be water quality issues utilising modelled data:

They don’t really [the farmers]—they don’t believe any of the data that has come out [indicating mystification] (A3).

It was also explained that the process of giving advice can be very difficult due to the lack of willingness to change practices that have occurred for multiple generations:

You don’t tell them what to do or how to farm, because obviously that’s what they’ve done for generations. (A1).

The mysterious nature of dealing with the 1622™WQ application was explicitly noted by two involved farmers but they indicated they were at least trying to utilise the application:

Actually now that you mention it, when it came out I think I tried to [access the app] and I couldn’t get there and I never went back to it [indicating mystification]. I’ll have to chase that up… and have another go at it (IF6).

Yeah, I downloaded it and I thought all this is—well, like I said before, instant world. I thought ‘am I missing a tab here’ and I’m going around and I couldn’t see anything so I went, oh well [indicating mystification] (IF2).

So the diversity of technological readiness—from low comfort through to resistance—indicated that although sugarcane farmers are diverse, most fell into the digi-MAST mode of mystery, as shown in the middle box of Fig. 2 (category 2 (M)). Similarly, these quotes point to the need to better integrate human and technological components across agricultural advisory service and farmer networks, which will be discussed in the following sections. For example, the following comments suggest that farmers tended to downplay their technical skills:

You’d have to because my telephone is smarter than I am [indicating mystification]. I’ve got no idea what I’m doing, to be honest (IF1).

I’ve got to tell you that I am very, very unskilled [indicating mystification] when it comes to computers (IF3).

The same interviewees later made comments found in the spark and transform modes of digi-grasping, however, all responses related to the ‘mystery of the digital world’ stemmed from farmers, both those involved and not involved with the P25 and DGBR initiatives and in reference to advisors perceptions about farmers.

### Awareness of the digital world (A)

Currently, agricultural advisory services’ and farmers’ interactions can be time consuming and costly, with travel to and from the sugarcane farm locations haphazard and at times bothersome for busy farmers. As such, Fig. 2 represents the current state of interactions 1 and 2 between humans and technologies on-farm with agricultural advisory services (in reference to the 1622™WQ application) both pre and post farm gate as ‘aware’. Farmers use agricultural advisory services when they are required to, can afford to, or have the time, but their interest in developing or transforming these interactions through technological means is currently limited due to a lack of value proposition. For example, two respondents are aware that this digital technology was available to use, although there remain barriers to uptake:

You explain to farmers how to use that technology, or you have someone in there who can help farmers use that technology… I just think the more apps [like 1622™WQ—indicating awareness] we can have out there, and it’s just got to be a matter of learning it and using it more often (NF5).

I went on the [1622™WQ app—indicating awareness] in the first week, having a look whether it’s updated, and I know [a researcher’s] been doing tests out here to make sure he can hook up to the network with rain gauges that aren’t in place yet. Yeah, the initial platform looks good (IF2).

The first couple of times I went on there [to the 1622™WQ app—indicating awareness] I brought up our area and it showed where the monitors were. I thought it was reasonably easy to use, except that I deleted it [somehow] (IF5).

The awareness of digital technologies is increasing with respect to bridging the gaps across different agricultural advisory services applications, which are likely to improve as further rounds of technological iteration occur. When considering the digi-MAST framework, these farmer-related interactions (1 and 2 in Fig. 3) could be argued to be holding back the digitalisation of the agricultural knowledge and advice network examined in this case. However, these interviewees provide justifications for their reasoning in terms of concerns about the implications of digital agriculture. Interviewees suggest the development of an industry network of learning and practice would support confidence and capability building in the digital agriculture space but that this network is not mature as yet, justifying previous work in different contexts (Carolan 2018; Fleming et al. 2018; Wolfert et al. 2017).

### A spark empowers human beings in the digital world (S)

While the first two modes of digi-grasping (mystery and aware) were eluded to through discussions with farmers, the spark and transform modes primarily included agricultural advisory service interviewee responses, suggesting agricultural advisory services were operating in different modes of digi-MAST. While the term ‘empowered’ was initially utilised in the digi-grasping descriptions of modes (Dufva and Dufva 2019), to make the framework easier to follow and pronounce we utilise ‘spark’ as a term more recognised in theories of innovation and management science (Sutton 2002). Spark also highlights the importance of stakeholder interest driving their learning which better described the Authors’ perceptions of this mode. Interactions between humans and technologies involving post-farm gate data and information were the subject of discussions whereby researchers and farmers found a spark of excitement, interest, and relevance in the 1622™WQ application. For example, while IF1 joked about their skills in managing digital devices (see mystery mode sub-section), their understanding of the monitoring technologies allowed them to articulate what would satisfy them in terms of project outcomes:

I would just hope the first thing that comes to mind is I’d hope that that science is—would stand up to peer review and that it is robust. That if the trailer—all the stuff behind the panels and the trailer delivers, that’s when I’m a happy man [indicating they were sparked by understanding the technological infrastructure] (IF1).

Similarly, while IF2 was not interested enough to repeatedly use the application due to a lack of relevant information being provided (see mystery mode sub-section), this interviewee indicated significant learning through the P25-Digiscape alliance work:

One thing that did become apparent out of all this is before we started on this 2 years ago or 3 years ago, there’s a couple of lessons I learnt… [it] takes a bloody long time to get accurate data [indicating they were sparked by understanding the technological infrastructure]. Everyone thinks it’s instantaneous—it’s not (IF2).

These farmers, while reporting aspects of the ‘mysterious’ digital world in the first mode section, also seem to be sparked by opportunities that the digital technologies might provide into their futures—technologies that deliver accurate and close to real-time data—this is even more evident when researcher responses are considered. Two examples highlight empowerment of researchers and the changes they can see will be required into the future from working with digital sensor networks that link into the 1622™WQ app:

I mean from my perspective currently the sensors that they’re using are too expensive to do the fine grain insights [i.e. comparing one site to another downstream and so on] that I think are necessary for the project [indicating they were sparked by understanding the technological infrastructure and it needed to go further]… [to say] personally ‘what’s my impact’ is a little bit harder to do with the current infrastructure (R1).

I don’t know if real time is the right word, but the sort of connectivity you could have with them nowadays with phones and websites and things like that… was still a bit of a learning experience for me [indicating they were sparked by understanding the technological infrastructure] (R2).

Our interview findings suggest that interviewees involved with the input and post-farm gate agricultural advisory service human and technological interaction in this case study (category 1 and 3 in Fig. 3)—by active participation in consideration and collaboration to attempt to provide water quality data in real-time through 1622™WQ—were aligned with the spark mode of digi-grasping.

### Transformation and intentional creation in the digital world (T)

The transform mode involves experiencing changes in thinking that lead to creative value (following changes in perceptions and behaviour) in the digital domain—similar to perspectives of transformation reported as an individual experiential change (Duncan et al. 2018). Importantly, one farmer reflected on the value of such work building digital agricultural technologies into the future, particularly in terms of the potential to change the way water quality monitoring that feeds into 1622™WQ was practiced in a way that brought people together through design:

Automated equipment that’s testing the water all the time, not done manually, which takes the human factor out of it. I think once that’s tried and proven that’s a plus [indicating thoughts of transformation through creation of new technology in the future]… You’d sort of look when it starts to rain and that sort of thing. As I say, it’s part of bringing everybody in together (IF3).

Agricultural advisory service interviewees recognised the scientific and creative potential of the digital technology design process, suggesting that the worlds of research and advice may have a greater capacity to transform at present. The process of application development was explained to continue to improve usefulness to users:

But this year we’ll also spend some time designing (1622™WQ) because it’s very clunky in terms of interaction and user experience [indicating thoughts of transformation through creation of new technology in the future]. So, we’ll redesign that into something that can basically be for future release (R1).

The transformation of the systems through which one agricultural advisory service interviewees’ working life was conducted was described in detail—to maximise temporal benefits:

We’ve gone from nearly a hundred per cent in bowls [manual sampling] to now real-time in situ probes with algorithms running over them and everything in between. I’ve got a bit of a vision where although traditional monitoring will never be replaced [indicating thoughts of transformation through creation of new technology in the future] you’ll always need that core monitoring validation point. We're going to wind down our traditional monitoring and ramp up our real-time (R5).

Primarily, it was engagement with agricultural advisory service interviewees that provided insights suggesting that, if they were not there already, they were very close to experiencing some dramatic changes to their everyday working lives in the context of developing, interacting with, and considering the 1622™WQ application. As such, Fig. 2 represents engagement between input and post-farm gate agricultural advisory services (interaction 3) as being at a stage whereby transformative changes are emerging in how these interviewees conduct their day-to-day lives. These results are caveated: situating the digi-MAST framework in such a social network recognises that individual human modes can fluctuate given feedbacks with others in the system and on the status of an individual actor’s perceptions concerning a specific technology at a given point in time in a given context. Crucially, the transformation here is not just about the technology or doing something in a new way, but about learning, engaging with others and new ways of thinking.

## Discussion: becoming a digi-MASTer on the quest for digital enlightenment

The digi-MAST framework provides a contribution to help understand complexity in terms of interactions between humans and digital technology and builds on existing four-pronged developmental heuristics—for example DIKW (Janssen et al. 2017) or critiques of the agricultural technology adoption model utilising aspects of propositions, encounters, dispositions, and responses (Glover et al. 2019). Conceptualising individual and collective movement through the digi-MAST modes in relation to specific technologies is important in part because the socio-technical transition to digitally enabled agricultural innovation systems will not occur in the short term (Fielke et al. 2019), nor will the benefits be equitably grasped by agricultural innovation system stakeholders (Jakku et al. 2019). As such, a mechanism for situating the mode of ‘grasp’ that individuals and collectives have on digital technologies in the present, as well as opportunities that can be created in the future, will be critical to managing digital agtech investment horizons and when weighing up costs against benefits of using a given technology or platform of technologies. The digi-MAST framework grounds notions of being and doing in the digital world, building on Ayre et al. (2019), to allow farmers and advisors to harness their capabilities to determine the value of specific digital agricultural technologies.

Due to the increasing connectivity and transparency future digital agricultural systems will likely involve (Lioutas et al. 2019), such a framework can help to relieve individual and collective anxieties about the unknown and embrace the process of, and aspirations to, transform human imagination (Dowd et al. 2014). The absorptive capacity, or ability to turn information into beneficial decision making (Turner et al. 2017), built through engagement with the technologies and individual learning through a design-process including the digi-MAST framework can be repeatedly and strategically drawn upon whenever new digital challenges to understanding present themselves (Calcagni et al. 2019; Rauschmayer et al. 2015). This framework will help research and development investments in digital technological processes to maximise their overall return on investment, utilising lessons from decision support tool adoption failures of decades passed, by allowing human actors to re-script their agricultural lives by bringing their agency to the human-centred design process (Rose et al. 2016, 2018). Should an individual or organisation choose not to partake in the digitalisation of agricultural systems this would be done by choosing to live with the mystery of digital world. Iterative and ongoing digital technological developments should allow for such diversity and be cognisant of the fact that some will actively choose to resist the intellectual and technical skill development required to reach the transform mode due to the costs and/or perceived risks involved. Transaction costs and data governance arrangements mean that not every digital technology is worth investing in for every farmer, advisor or researcher (Glover et al. 2019; Wiseman et al. 2019). For example, farmers may choose to consciously shun digitalisation on their farm altogether, or specific forms of digital technologies, in an act of defiant choice. Either way, it is hard to see the opportunity to pursue a more digitally enlightened future, through agriculture 4.0 or otherwise, disappearing any time soon (Helbing 2019).

As noted in the method, the results of these interviews informed 1622™WQ project planning workshops with members of the DGBR team where the digi-MAST framework was utilised to reflexively question the status (readiness, curiousness, and willingness) of each of the relevant stakeholder groups in terms of the tools being developed (Fielke et al. 2017; Pant and Odame 2017). This real-time monitoring, evaluation and learning process explicitly highlighted the shifts in human-digital interactions required by the stakeholder groups if certain outcomes were to be achieved and how future foci and aims might need to be altered if certain impacts were to be realised (Stone-Jovicich et al. 2019). Regarding the sugarcane case study, movement through the modes of digi-MAST beyond mystery and aware in those categories and interactions in Fig. 3 will be required to achieve a future state that could be labelled ‘responsible agriculture 4.0’ (Rose and Chilvers 2018). The knowledge and ability to shift the mindsets of individual farmers, advisors and researchers will take time and effort spent interacting with (and through) digital technologies—supporting previous assertions that typical socio-technical transition time frames of a human lifetime (give or take) will be required (Fielke et al. 2019). We propose, however, that it is possible that real-time data can help farmers establish the link between riverine discharge and N concentrations and the objective of the 1622™WQ app was to increase farmer awareness.

The digi-MAST framework can be applied across interactions and feedbacks associated with human learning processes so that actors can become familiar with and potentially understand how to obtain value from new technologies like 1622™WQ. We argue such a framework will be critical to grasp agriculture 4.0—to turn individual or collectively perceived agricultural imaginations into individual or collectively perceived agricultural facts and/or realities. The digi-MAST framework, due to a grounding in the agricultural innovation system conceptual tradition, also allows for consideration of higher-level implications of the digital turn (Fielke et al. 2018; Hall et al. 2003; Klerkx et al. 2012; Knickel et al. 2009). For example, the suite of digital technologies broader society is now engaged with have been argued to have changed traditional models of the accumulation of wealth leading to ‘surveillance capitalism’ (Zuboff 2019). The digi-MAST framework, applied to the example of 1622™WQ, captures valid concerns from farmers regarding their perceptions of their vulnerability in the face of digital agricultural technologies, similar to previous work in the grains sector of Australia (Jakku et al. 2019). The expectation of farmers, advisors and researchers to adopt new digital technologies needs to be more fairly incentivised if these stakeholders are to realise the benefits of such technological change (Glover et al. 2019). Is it really surprising that sections of the agricultural community remain critical when there are already examples of disruptive, technologically-driven corporations utilising operating procedures whereby their digital architectures evolve by-design to serve economically powerful interests with private business models that bypass user awareness (Zuboff 2019)?

To begin to address some of the limitations of this briefcase study report, future research will endeavour to utilise this framework to test and validate (or not) its utility by exploring perceptions in other agricultural knowledge and advice networks and in the context of different digital technological use cases. Such work will build understanding of the mechanisms with which respondents are open to shift between categories, for example from aware to spark or spark to transform (and vice versa). Similarly, the capabilities and processes individuals might build grasping different digital technologies over time will likely lead to an increase in efficiency of digi-MAST mode traversal, leading to the development of skilled human digi-MASTers. The shift to digital interaction forced by the COVID-19 pandemic also provides impetus for change. Appropriate pedagogical approaches to increase digital awareness and spark farmers interests will also follow. Ultimately this work will provide a platform to allow individual farmers to find their niche in future agricultural systems through the ongoing testing of technological engagement through the innovation process (Berthet and Hickey 2018; Fielke et al. 2018).

## Conclusion

This paper aimed to analyse the development of a case study digital agricultural technology (1622™WQ) within a specific Australian sugarcane farming agricultural knowledge and advice network. We interpreted existing literature to create the digi-MAST framework, which was inductively tested against interview data from farmers and agricultural advisory service stakeholders working to increase awareness of the water quality implications of on-farm practice as a means of encouraging change in thinking and behaviour. Through our case study, we demonstrated that different individual human actors are at different stages of their digitalisation journeys in relation to the specific technology being developed (1622™WQ) and other digital technologies more broadly. From farmers being mystified and preferring to keep it that way, through to evidence of sparks and some agricultural advisory service stakeholders seeking to transform their everyday practice through intentional creation and iteration of digital technological developments. We argued that digital technological developments will allow for such change, understanding that there exist those that will actively choose to resist using their limited resources (time, money, energy) to develop the skills required to reach a consciously digitally enlightened state. It is a big ask to risk your livelihood by trusting the technological ecosystem behind networked digital agricultural technology platforms. In this case study, the digi-MAST framework has been embraced by the DGBR team as a useful device that accompanied social science and human-centred design workshop sessions. The framework has also helped qualify potential social risks regarding a divide between actors’ expectations to contribute to the digital technology development project for this specific agricultural knowledge and advice network.

## Footnotes

1 Approved by CSIRO Social Science Human Research Ethics Committee—project 106/17.

2 For more information, the interested reader is referred to Vilas et al. (2020) for details on the interactions that led to the development of the application.
